# The role of trimethoprim/sulfamethoxazole in preventing opportunistic infections in systemic lupus erythematosus patients receiving low-level immunosuppressive treatment: an open-label, randomized, controlled trial

**DOI:** 10.1007/s10238-024-01503-z

**Published:** 2024-10-19

**Authors:** Paopat Munthananuchat, Pintip Ngamjanyaporn, Prapaporn Pisitkun, Porpon Rotjanapan

**Affiliations:** 1 Department of Medicine, Faculty of Medicine Ramathibodi Hospital, Mahidol University, Bangkok, Thailand; 2 Division of Allergy, Immunology, and Rheumatology, Department of Medicine, Faculty of Medicine Ramathibodi Hospital, Mahidol University, Bangkok, Thailand; 3 Division of Infectious Diseases, Department of Medicine, Faculty of Medicine Ramathibodi Hospital, Mahidol University, 270 Rama VI Road, Ratchatewi, Bangkok, 10400 Thailand

**Keywords:** SLE, Trimethoprim/sulfamethoxazole, Opportunistic infections, Low-level immunosuppression

## Abstract

**Objective:** Systemic lupus erythematosus (SLE) patients receiving immunosuppressive therapy are at risk for opportunistic infections (OIs), particularly *Pneumocystis* pneumonia (PCP). This study aimed to evaluate the effectiveness of trimethoprim/sulfamethoxazole (TMP/SMX) as primary prophylaxis against OIs and its adverse effects in SLE patients receiving low-level immunosuppressive treatment in a real-world setting. **Methods:** This open-label randomized controlled trial enrolled SLE patients receiving low-level immunosuppressive treatment at Ramathibodi Hospital between May 2021 and December 2022. Patient demographics and relevant clinical data were collected. Participants were randomized 1:1 to receive TMP/SMX or no prophylaxis, with dose adjustments according to renal function. The incidences of TMP/SMX-sensitive OIs and adverse events were monitored for 12 months post-enrollment. **Results**: The trial was terminated early due to a high rate of adverse drug reactions (ADRs) associated with TMP/SMX. In total, 138 SLE patients receiving low-level immunosuppressive treatment were enrolled. Most patients (98.4%) were in disease remission. No TMP/SMX-sensitive OIs were observed in either group during the 12-month follow-up period. Among individuals receiving TMP/SMX, 10/70 (14.3%) developed ADRs. Of these 10 patients, eight experienced grade 1 ADRs, and two had grade 3 ADRs; all declined to resume prophylaxis. There were no deaths in the study. **Conclusions**: During the 12-month follow-up period, no TMP/SMX-sensitive OIs occurred in SLE patients receiving low-level immunosuppressive therapy, suggesting that primary prophylaxis with TMP/SMX may not significantly benefit this population. The high rate of ADRs observed underscores the need for clinicians to carefully consider the risks and benefits of TMP/SMX prophylaxis in these patients.

## Introduction

Opportunistic infections (OIs), particularly *Pneumocystis* pneumonia (PCP) and nocardiosis, are well-recognized complications in individuals with acquired immunodeficiency syndrome (AIDS), especially when CD4 counts drop below 200 cells/mm^3^. Importantly, nonhuman immunodeficiency virus (HIV)-infected patients receiving immunosuppressive therapy, such as those with connective tissue disease, solid or hematologic malignancies, or organ transplants, are also at risk for OIs [1].

Systemic lupus erythematosus (SLE) is a connective tissue disease involving multiple organs with high prevalence in the Thai population. Immunosuppressive therapy is the primary treatment used to improve clinical outcomes. However, several studies have linked OIs to increased hospitalizations and higher mortality rates in non-HIV patients receiving immunosuppressive treatments, particularly high-dose corticosteroids (≥ 30 mg/day prednisolone equivalent) [2, 3]. Using Taiwan’s National Health Insurance data for 1997 to 2010, Yang et al. conducted a cohort study to investigate whether the incidence of OIs differed among SLE patients who received different doses of corticosteroids. The index day was defined as 3 months after the initial SLE diagnosis. A nonsteroid cohort was matched 4:1 with the steroid cohort according to age, sex, and index day. The study results showed that the cumulative incidence of OIs, including PCP, tuberculosis, and herpes zoster infection, increased according to corticosteroid dose. The adjusted hazard ratios for OI were 1.40 (95% confidence interval [CI] 0.78–2.51) in the low-dose corticosteroid group (7.5–15 mg/day prednisolone equivalent), 1.72 (95% CI 1.02–2.91) in the medium-dose corticosteroid group (15–30 mg/day prednisolone equivalent), and 1.96 (95% CI 1.17–3.28) in the high-dose corticosteroid group (> 30 mg/day prednisolone equivalent) [4]. A previous study of rheumatic diseases revealed PCP in patients receiving prolonged corticosteroid doses of < 30 mg/day prednisolone equivalent; significant risk factors included baseline lymphopenia and concomitant pulse corticosteroid treatment [5].

Trimethoprim/sulfamethoxazole (TMP/SMX) is a cost-effective antimicrobial with a broad range of US Food and Drug Administration-approved indications, including acute infective exacerbation of chronic bronchitis, pediatric otitis media, traveler’s diarrhea, urinary tract infections, shigellosis, and PCP. In the pre-antiretroviral therapy era, TMP/SMX played a key role in primary prophylaxis for PCP among AIDS patients, in whom it significantly reduced the incidence of PCP [1]. Additionally, TMP/SMX is used as prophylaxis and treatment for toxoplasmosis [6]. TMP/SMX continues to be used as primary prophylaxis for PCP in HIV patients with a CD4 count of < 200 cells/µL at a dose of one single-strength (80/400 mg) tablet daily. Prophylaxis discontinuation is appropriate when the CD4 count exceeds 200 cells/µL for 3 months [7]. Recommendations regarding the use of TMP/SMX prophylaxis in non-HIV patients have been primarily based on limited clinical evidence. In 2016, the 5th European Conference on Infections in Leukaemia established evidence-based recommendations for PCP prophylaxis in non-HIV-infected patients with underlying hematological conditions, including allogeneic hematologic stem cell transplant recipients. These recommendations suggest a dose of either one single-strength (80/400 mg) tablet daily or one double-strength (160/800 mg) tablet daily or thrice weekly. Discontinuation criteria for PCP prophylaxis in patients with recovering immunity have not been defined, and decisions are made on an individual basis [8]. A review by Salzer et al. concerning treatment and prophylaxis in non-HIV immunocompromised patients suggested considering prophylaxis in non-HIV immunocompromised patients with a combination of risk factors, including age, pulmonary comorbidity, and a corticosteroid dose of > 15 mg/day. There were no recommendations on prophylaxis in patients receiving a combination of immunosuppressive therapy. Moreover, for non-HIV patients with connective tissue diseases receiving high-dose immunosuppressive treatment, there are no criteria for de-escalating or discontinuing TMP/SMX primary prophylaxis during disease remission or when immunosuppressive therapy is tapered. In the real-world setting, some clinicians prefer to prescribe TMP/SMX prophylaxis to most patients who receive the combination of immunosuppressive therapy, given that there have been no solid clinical studies to address the actual immune status of each individual during maintenance treatment. This lack of guidance extends to non-HIV patients receiving low-level immunosuppressive regimens, who may exhibit an increased risk of infection due to combination therapies [7].

Considering this knowledge gap, we hypothesized that TMP/SMX primary prophylaxis is not necessary for patients receiving the combination of low-level immunosuppressive regimens.

## Objectives

The primary objective of this study was to evaluate the role of TMP/SMX primary prophylaxis in SLE patients receiving low-level immunosuppressive therapy. The secondary objectives were to assess the incidence of TMP/SMX-sensitive OIs in SLE patients receiving low-level immunosuppressive treatment and to evaluate adverse drug reactions (ADRs) among these patients.

## Methods

### Study design

This open-label randomized controlled trial was conducted at Ramathibodi Hospital from May 2021 to December 2022. SLE patients aged ≥ 18 years diagnosed using the 2019 European League Against Rheumatism/American College of Rheumatology Classification Criteria for Systemic Lupus Erythematosus (EULAR/ACR) were screened for eligibility. [9] Patient demographics, SLE Disease Activity Index 2000 (SLEDAI-2 K) scores, SLE treatment regimens, and other relevant data were collected. Enrolled patients were randomized 1:1 to receive TMP/SMX or no prophylaxis using a computer-generated block randomization scheme. The TMP/SMX dose was adjusted based on renal function. The incidence of TMP/SMX-sensitive OIs was monitored for 12 months post-enrollment in all patients, and adverse events were assessed during the study period. Patients already receiving TMP/SMX discontinued the medication for 2 weeks before enrollment.

Patients were excluded from the study if they met any of the following criteria: receipt of immunosuppressive therapy other than a low-level regimen at enrollment, history of sulfa or TMP/SMX allergy, history of TMP/SMX-sensitive OIs in the preceding 6 months, history of high-dose pulse immunosuppressive therapy within the preceding 6 months, SLE with overlap syndrome, solid or hematologic cancer, pregnancy, solid or hematologic organ transplant recipient, HIV infection, history of cytomegalovirus infection in the preceding 3 months, and/or receipt of dapsone or pentamidine in the previous month.

## Definitions

### High- and low-level immunosuppressive regimens

The levels of immunosuppressive regimens in this study were adapted from the 2013 Infectious Diseases Society of America clinical practice guideline for vaccination of an immunocompromised host. Regimens were categorized as single-drug or combination, then subclassified as low-level or high-level [10].

### Single-drug regimens

Low-level immunosuppressive therapy was defined as prednisolone < 20 mg/day (or equivalent) for ≥ 14 days or alternate-day corticosteroid therapy, methotrexate (MTX) < 0.4 mg/kg/week, azathioprine < 2 mg/kg/day, 6-mercaptopurine (6-MP) < 1.5 mg/kg/day, mycophenolate mofetil < 1.5 g/day, mycophenolic acid < 1,080 mg/day, and any other immunosuppressive regimen not classified as high-level.

High-level immunosuppressive therapy was defined as prednisolone ≥ 20 mg/day (or equivalent) for ≥ 14 days, MTX > 0.4 mg/kg/week, azathioprine > 2 mg/kg/day, 6-MP > 1.5 mg/kg/day, biologic immune modulators (e.g., tumor necrosis factor-alpha blockers and rituximab), mycophenolate mofetil > 1.5 g/day, and mycophenolic acid ≥ 1,080 mg/day.

### Combination drug regimens

Low-level immunosuppressive therapy was defined as the use of two immunosuppressive drugs (e.g., MTX, azathioprine, 6-MP, mycophenolate mofetil, or mycophenolic acid) at doses low-level as defined above, or the combination of prednisolone with one of the above immunosuppressive drugs at low-level doses. High-level immunosuppressive therapy was defined as the use of ≥ 3 drugs, including prednisolone or any immunosuppressive drugs listed above, at any dose.

### Definitions of TMP/SMX-sensitive OIs

TMP/SMX-sensitive OIs were defined as infections more frequently diagnosed in immunocompromised individuals, including PCP, toxoplasmosis, nocardiosis, and other diseases caused by pathogens susceptible to TMP/SMX in culture [11].

#### PCP

1. Definite PCP was defined as the detection of *Pneumocystis jirovecii* by histopathology staining or polymerase chain reaction (PCR) with relevant clinical manifestations and compatible chest radiograph findings (e.g., ground-glass appearance and interstitial pattern).
2. Probable PCP was defined as the presence of relevant clinical manifestations and chest radiograph findings without histopathology staining-based or PCR-based evidence of PCP, combined with favorable outcomes after TMP/SMX treatment.
3. Colonization by *P. jirovecii* was defined as the detection of PCP solely by PCR analysis of respiratory specimens without relevant clinical manifestations, chest radiograph findings, or histopathology staining-based confirmation [12].

#### Nocardiosis

1. Definite nocardiosis was defined as the isolation of *Nocardia* spp. from clinical specimens using conventional culture methods, combined with relevant clinical manifestations and radiographic findings (e.g., pulmonary consolidation, pulmonary abscess, or brain abscess).
2. Probable nocardiosis was defined as the identification of *Nocardia* spp. in clinical specimens by Gram staining and/or modified acid-fast staining, combined with relevant clinical manifestations and radiographic findings (e.g., pulmonary consolidation, pulmonary abscess, or brain abscess).
3. Possible nocardiosis was defined as the presence of relevant clinical manifestations and radiographic findings (e.g., pulmonary consolidation, pulmonary abscess, or brain abscess) without available clinical specimen data, combined with favorable outcomes after nocardiosis treatment [13].

#### Toxoplasmosis

1. Definite toxoplasmosis was defined as the identification of *Toxoplasma gondii* by PCR analysis of clinical specimens combined with relevant clinical manifestations and radiographic findings (e.g., multiple parenchymal nodular lesions or ring-enhancing lesions in the brain).
2. Possible toxoplasmosis was defined as the presence of relevant clinical manifestations and findings (e.g., multiple parenchymal nodular lesions or ring-enhancing lesions in the brain) without confirmation of *Toxoplasma gondii* in clinical specimens, combined with favorable outcomes after toxoplasmosis treatment [14].

### TMP/SMX dosage regimens

The dosage of TMP/SMX for primary prophylaxis was adjusted according to renal function, determined by the Chronic Kidney Disease Epidemiology Collaboration (CKD-EPI) equation for estimated glomerular filtration rate (eGFR). Patients with an eGFR > 30 ml/min/1.73 m^2^ received oral TMP/SMX (80/400 mg), two tablets once daily; those with an eGFR between 15 and 30 ml/min/1.73 m^2^ received oral TMP/SMX (80/400 mg), one tablet once daily; and those with an eGFR < 15 ml/min/1.73 m [2] received oral TMP/SMX (80/400 mg), one tablet three times per week [15].

### ADRs

ADRs were classified and graded using the Common Terminology Criteria for Adverse Events (CTCAE) version 5.0.

### Statistical analysis

The sample size was calculated for a randomized controlled trial with binary data, using a PCP incidence of 7.6% among SLE patients not receiving TMP/SMX prophylaxis, as reported by Vananuvat et al. [16] The target sample size was 248, allowing for a 20% dropout rate. Type I and II error rates were set at 0.05 and 0.2, respectively.

Data were analyzed using Stata version 17.0 software. Baseline characteristics and outcomes were compared between groups using the Chi-square test for categorical data and the Mann–Whitney U test or Student’s t test for continuous data. The threshold for statistical significance was defined as *p* < 0.05.

## Results

### Patient characteristics

**Fig. 1:**
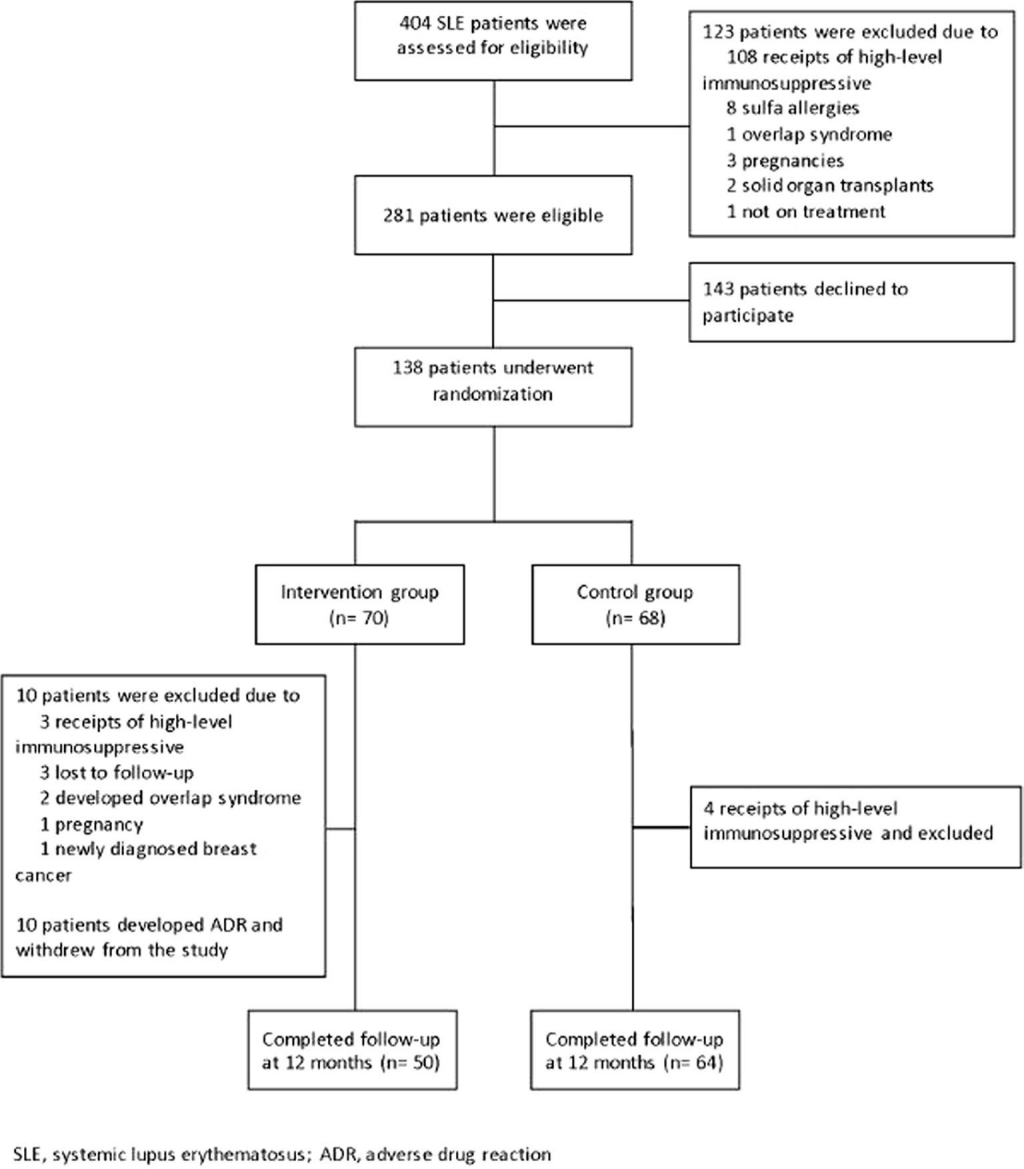
Protocol flow chart

Of 404 SLE patients screened, 123 were excluded due to high-level immunosuppressive treatment (*n *= 108), sulfa drug allergy (*n* = 8), pregnancy (*n* = 3), history of solid organ transplantation (*n* = 2), overlap syndrome (*n* = 1), or palliative care (*n* = 1). Of the remaining 281 eligible patients, 143 declined to participate. Thus, 138 patients were randomized to the intervention (*n* = 70) and control groups (*n* = 68), as shown in Fig. 1.

During the study, 10 patients in the intervention group were excluded for the following reasons: a need for high-level immunosuppression (*n* = 3), loss to follow-up (*n* = 3), overlap syndrome (*n* = 2), pregnancy (*n* = 1), and breast cancer diagnosis (*n* = 1). Additionally, 10 patients in this group discontinued TMP/SMX due to ADRs. In the control group, four patients were excluded due to increased disease activity requiring high-level immunosuppression.

Ultimately, 125 patients completed the study (61 and 64 in the intervention and control groups, respectively). Baseline characteristics are presented in Table 1. The mean ages were 45.4 (12.9) years in the intervention group and 45.4 (12.8) years in the control group; most patients were women (57 of 61 [93.4%] in the intervention group and 60 of 64 [93.8%] in the control group.) Most patients had a SLEDAI-2 K score < 4 in both groups. Median times since SLE diagnosis were 11.1 years (2.0–18.3) in the intervention group and 11.6 years (6.0–20.5) in the control group. Comorbidities, including chronic kidney disease, cardiovascular disease, and diabetes mellitus, were comparable between groups. Prednisolone was the most commonly prescribed immunosuppressive drug, taken by 51 of 61 patients (83.6%) in the intervention group and 47 of 64 patients (73.4%) in the control group. Forty-six of 61 patients (75.4%) in the intervention group and 39 of 64 patients (60.9%) in the control group received a combination immunosuppressive regimen (*p* = 0.083). The median (interquartile range) corticosteroid dose was 5 mg (2.5–7.5) prednisolone per day in both groups. No TMP/SMX-sensitive OIs were observed in either group during the 12-month follow-up period, and no deaths were reported.

**Table 1:** Baseline characteristics of intervention and control groups

Variables	Intervention group (n = 61)	Control group (n = 64)	*p*-value
Age, mean (SD), years	45.2 (12.9)	45.4 (12.8)	0.991
Sex, female, n (%)	57 (93.4%)	60 (93.8%)	1.000
Time since SLE diagnosis, median (range), years	11.1 (2.0–18.3)	11.6 (6.0–20.5)	0.412
BMI, mean (SD), kg/m^2^	23.7 (5.6)	23.7 (5.9)	0.315
ALC, mean (SD), cells/mm^3^	1775.9 (857.5)	1588.0 (693.9)	0.180
CD4, median (range), cells/mm^3^	587.5 (430.0–783.0)	483.5 (322.5–749.0)	0.296
SLEDAI-2 K, < 4, n (%)	60 (98.4%)	63 (98.4%)	1.000
TST, positive, n (%)	59 (96.7%)	63 (98.4%)	0.613
eGFR, mean (SD), ml/min/1.73 m^2^	97.8 (27.6)	96.1 (30.7)	0.750
Preexisting conditions, n (%)			
DM	2 (3.3%)	2 (3.1%)	1.000
CVS	2 (3.3%)	3 (4.7%)	1.000
CKD	5 (8.2%)	5 (7.8%)	1.000
Immunosuppressive treatment, n (%)			0.083
Monotherapy	15 (24.6%)	25 (39.1%)	
Combination therapy	46 (75.4%)	39 (60.9%)	
Prednisolone, n (%)	21 (67.7%)	13 (56.5%)	0.167
Median dose (IQR), mg/day	5 (5–5)	5 (2.5–7.5)	0.270

*TST* tetanus skin test, *BMI* body mass index, *ALC* absolute lymphocyte count, *eGFR* estimated glomerular filtration rate, *DM* diabetes mellitus, *CVS* cardiovascular syndrome, *CKD* chronic kidney disease, *IQR* interquartile range, *SD* standard deviation, *SLEDAI-2 K* Systemic Lupus Erythematosus Disease Activity Index 2000

### ADRs

A comprehensive review of drug-associated hypersensitivity reactions was performed, and no other culprit drugs or conditions could explain the events. Patient enrollment was terminated early due to a high rate of ADRs associated with TMP/SMX, as reported to the Ethics Committee. Ten of 70 patients (14.3%) receiving TMP/SMX primary prophylaxis in the intervention group developed ADRs (Table 2). Half of these patients experienced skin reactions, all developing at an average of 1 month after the first dose of TMP/SMX. These reactions included one drug rash with eosinophilia and systemic symptoms (grade 3); other skin reactions were minor (grade 1–2). One patient experienced grade 3 hepatitis after 2 months of TMP/SMX prophylaxis; four patients had minor gastrointestinal side effects (grade 1) after 3–6 months. All participants with ADRs fully recovered after conservative treatment except for one patient who had grade 3 skin reaction and received a 4-week course of a higher dose of prednisolone and documented a complete recovery after that. All participants who experienced ADRs chose to withdraw from the study.

**Table 2:** Adverse drug reactions caused by trimethoprim/sulfamethoxazole in the intervention group

Adverse drug reactions	Number of cases	Onset (months)
Gastrointestinal side effects	4	3.2–5.6
Skin rash	4	0.4–1.2
LFT abnormality	1	1.9
*Serious adverse drug reactions*		
Drug rash with eosinophilia and systemic symptoms	1	0.5

*LFT* Liver function test

## Discussion

SLE is a complex autoimmune disease with diverse clinical features. SLE pathophysiology is characterized by the production of multiple autoantibodies, leading to immune complex formation, deposition, and other manifestations of immune dysregulation [17]. The global incidence of SLE is estimated to be 5.14 (range, 1.4–15.13) per 100,000 person-years, and the disease predominantly affects women. In the Asia–Pacific region, the crude incidence ranges from 0.9 to 3.1 per 100,000 person-years, and the mean age of onset ranges from 25.7 to 34.5 years. Previous studies have shown that SLE is associated with high annual hospitalization costs, particularly during periods of disease activity [18–20].

The treatment of SLE consistently relies on immunosuppressive agents. More aggressive treatment is necessary during active disease, whereas less intensive maintenance therapy can be considered when the disease is controlled. Drugs commonly prescribed during maintenance therapy include prednisolone, rituximab, mycophenolic acid, tacrolimus, and azathioprine. However, these immunosuppressive regimens can impair humoral and cell-mediated immune responses, increasing susceptibility to OIs. Impaired humoral immune responses predispose patients to bacterial infections, whereas impaired cell-mediated immune response increases susceptibility to intracellular pathogens (e.g., *Candida* spp., *Cryptococcus* spp., *P. jirovecii*, and *T. gondii*). Due to the heightened risk of infection, preventative measures such as influenza vaccination and screening and prophylaxis for chronic and opportunistic infections are recommended. The 2022 European Alliance of Associations for Rheumatology (EULAR) recommends PCP prophylaxis for adults with autoimmune inflammatory rheumatic diseases receiving high-dose glucocorticoids (15–30 mg prednisolone or equivalent) for more than 2–4 weeks. The suggested TMP/SMX dose is one single-strength (80/400 mg) tablet daily or one double-strength (160/800 mg) tablet three times weekly [21, 22]. A large retrospective cohort study by Park et al., involving 28,292 treatment episodes with non-high-dose steroids, evaluated the use of TMP/SMX as primary prophylaxis for PCP in patients with rheumatic diseases. The findings indicated that the incidence of PCP was relatively low in patients receiving low to medium corticosteroid doses (prednisolone equivalent < 15 mg/day) compared with patients in the high-dose corticosteroid group (prednisolone equivalent ≥ 15 mg/day) and patients with additional risk factors receiving moderate doses. Moreover, Qian et al. identified renal dysfunction and lymphopenia as independent risk factors for PCP infection in children with SLE receiving prolonged high-dose steroid therapy [5, 23].

Although TMP/SMX prophylaxis is recommended for HIV-negative immunocompromised patients receiving high-dose corticosteroids, there is no consensus regarding its use in patients receiving lower-intensity immunosuppressive regimens or a combination of low-level immunosuppressive agents, including other specific non-corticosteroid immunosuppressive drugs. Furthermore, there is no evidence-based guidance concerning the appropriate timing for prophylaxis discontinuation [7].

In the present study, most patients were women and had low SLEDAI-2 K scores. All patients received low-level immunosuppressive regimens; half received combination immunosuppressive therapy, and the median prednisolone dose was 5 mg/day. Notably, no TMP/SMX-sensitive OIs occurred during the 12-month follow-up period. However, the intervention group experienced a relatively high rate of ADRs, mostly mild to moderate skin and gastrointestinal manifestations. One patient developed a serious ADR, which fully resolved after treatment. Therefore, in the previous study that was performed before the HIV infection epidemic, reported reactions associated with sulfa prescriptions among hospitalized patients from 1966 to 1980 were 8% [24].

However, the relatively high rate of ADRs, including one serious but resolved ADR, raises concerns about the risks of unnecessary prophylaxis. Notably, ADR rates in our study are consistent with findings from Watanabe et al., who reported an incidence of drug hypersensitivity reactions ranging from 20 to 38% in SLE patients, particularly with sulfa antibiotics. These hypersensitivity reactions may result from shared pathogenic pathways between SLE and drug-induced eruptions, including mechanisms involving neutrophil extracellular traps (NETs) and T-cell activation. Imbalances in NET production and degradation, particularly in active SLE, have been associated with reduced NET clearance and enhanced necrotic skin reactions. Izuka et al. reported that specific antibodies, such as anti-Sm, anti-ribonucleoprotein (RNP), and anti-Ro/SS-A, were significantly associated with drug hypersensitivity in SLE. However, this finding requires confirmation because of the small sample size [25–29].

From an antibiotic stewardship and safety perspective, TMP/SMX prophylaxis might not be required for patients receiving low-level immunosuppressive therapy and may lead to ADRs. A previous study showed that unnecessary TMP/SMX use could disrupt the vaginal microbiota, potentially increasing the risk of urinary tract infections in SLE patients [30]. Thus far, there have been no reports of increased resistance to TMP/SMX among previously susceptible pathogens. However, although no resistance has been reported so far, the frequent and unnecessary use of antibiotics can contribute to the global problem of antimicrobial resistance. Overusing broad-spectrum antibiotics like TMP/SMX can promote resistance in targeted pathogens and the commensal microbiota, which serves as a reservoir for resistance genes. This raises concerns that, even in the absence of immediate resistance in TMP/SMX-sensitive organisms, the selective pressure exerted by unnecessary prophylaxis could contribute to the emergence of resistant strains over time [31, 32].

A notable strength of this randomized controlled study was that it highlighted the potential risk of prescribing unnecessary TMP/SMX prophylaxis to SLE patients receiving low-level immunosuppressive treatment in a real-world setting that this knowledge gap has never been addressed before in any clinical practice guidelines to our knowledge. The clinical relevance is enhanced by the absence of TMP/SMX-sensitive OIs during the 12-month follow-up period and the use of diverse immunosuppressive regimens beyond prednisolone alone. Patients with SLE can remain in a good state of health by consuming a well-balanced diet and regular exercise. Stress reduction, avoiding direct sunlight exposure, receiving regular comprehensive cardiovascular assessments to detect left ventricular diastolic dysfunction early, receiving necessary vaccinations per healthcare providers, and avoiding unnecessary antibiotic use [33–35]. However, this study had some significant limitations that should be considered when interpreting the results:

1. Small sample size**:** The study was prematurely terminated due to high ADR rates, reducing its statistical power. This limited our ability to detect rare OIs, and future studies should aim for larger sample sizes to provide more robust conclusions.
2. Single-center design: Conducting the trial at a single institution limits the generalizability of the findings, as patient demographics and clinical practices may differ in other settings. Multicenter trials would improve the external validity of such research.
3. Short follow-up duration: A 12-month follow-up may not capture long-term OI development, as some opportunistic infections can take longer to manifest. Longer follow-up periods in future studies beyond 12 months would provide a more comprehensive evaluation of TMP/SMX prophylaxis and a better assessment of long-term infection rates and ADRs.
4. Homogeneous study population: Most of our patients were in disease remission, which may not reflect the broader SLE population, particularly those with higher disease activity and non-Asian population. Future studies on a more diverse population would provide a more comprehensive understanding of TMP/SMX prophylaxis in SLE patients.
5. Lack of pharmacogenomic and comprehensive immune status evaluation: Including pharmacogenomics testing and monitoring of immune function, particularly lymphocyte subset or immunoglobulin levels, could have provided more profound insights into patient susceptibility to OIs or ADRs. This personalized approach could optimize prophylactic strategies.
6. The lack of a placebo group for comparison may have affected the study’s ability to assess subtle prophylactic effects and ADRs.
7. Definitions of low-level immunosuppressive therapy: Clinical practices and treatment regimens may differ. Therefore, a careful interpretation of the study results should be considered.

In conclusion, our findings indicate that TMP/SMX-sensitive OIs are not a significant concern for SLE patients on low-level immunosuppressive therapy, and the relatively high rate of ADRs suggests that routine prophylaxis may not be necessary in this population. Future research should explore the long-term safety of TMP/SMX, particularly in populations with higher disease activity, and consider incorporating pharmacogenomic and immunological assessments to personalize prophylactic strategies.

## Data Availability

Data are provided within the manuscript or supplementary information files.
